# Impact of Sox9 Dosage and Hes1-mediated Notch Signaling in Controlling the Plasticity of Adult Pancreatic Duct Cells in Mice

**DOI:** 10.1038/srep08518

**Published:** 2015-02-17

**Authors:** Shinichi Hosokawa, Kenichiro Furuyama, Masashi Horiguchi, Yoshiki Aoyama, Kunihiko Tsuboi, Morito Sakikubo, Toshihiko Goto, Koji Hirata, Wataru Tanabe, Yasuhiro Nakano, Haruhiko Akiyama, Ryoichiro Kageyama, Shinji Uemoto, Yoshiya Kawaguchi

**Affiliations:** 1 Department of Surgery, Kyoto University Graduate School of Medicine, Kyoto, Japan; 2 Department of Orthopaedics, Gifu University, Gifu, Japan; 3 Department of Clinical Application, Center for iPS cell Research and Application, Kyoto University, Kyoto, Japan; 4 Institute for Virus Research, Kyoto University Graduate School of Medicine, Kyoto, Japan; 5 Department of Gastroenterology and Hepatology, Kyoto University Graduate School of Medicine, Kyoto, Japan

## Abstract

In the adult pancreas, there has been a long-standing dispute as to whether stem/precursor populations that retain plasticity to differentiate into endocrine or acinar cell types exist in ducts. We previously reported that adult Sox9-expressing duct cells are sufficiently plastic to supply new acinar cells in *Sox9-IRES-CreERT2* knock-in mice. In the present study, using *Sox9-IRES-CreERT2* knock-in mice as a model, we aimed to analyze how plasticity is controlled in adult ducts. Adult duct cells in these mice express less Sox9 than do wild-type mice but Hes1 equally. Acinar cell differentiation was accelerated by Hes1 inactivation, but suppressed by NICD induction in adult Sox9-expressing cells. Quantitative analyses showed that Sox9 expression increased with the induction of NICD but did not change with Hes1 inactivation, suggesting that Notch regulates Hes1 and Sox9 in parallel. Taken together, these findings suggest that Hes1-mediated Notch activity determines the plasticity of adult pancreatic duct cells and that there may exist a dosage requirement of Sox9 for keeping the duct cell identity in the adult pancreas. In contrast to the extended capability of acinar cell differentiation by Hes1 inactivation, we obtained no evidence of islet neogenesis from Hes1-depleted duct cells in physiological or PDL-induced injured conditions.

During organogenesis, the plasticity of embryonic cells gradually decreases as lineage separation proceeds and cells differentiate into mature cell types. However, the generation of iPS cells and the direct reprogramming of some cell types into others clearly show the astonishing plasticity that is retained in adult cells^1^,^2^. The reprogramming can be created by artificially introducing a few transcription factors, and the plasticity of adult cells is shown in several physiological and pathological conditions, including organ maintenance, tissue regeneration and carcinogenesis. Indeed, organ-specific stem/progenitor cells have been identified in adult organs that continuously supply new cells, such as the skin and gut, where they maintain physiological organ homeostasis^3^,^4^. Other reports have shown the dedifferentiation of mature cells into an immature status during the regeneration process after injury^5^,^6^,^7^. In addition, pathological metaplasia of mature cell types sometimes causes malignant transformation^8^,^9^,^10^. However, in contrast with our understanding of the cell differentiation machinery during embryonic stages, details of the mechanism that controls adult cell plasticity *in vivo* largely remain to be elucidated.

There has been long-standing debate as to whether physiologically functioning stem/progenitor cell populations exist in the adult ductal compartment of the pancreas^11^. Several lineage-tracing experiments have been conducted to follow the fate of adult pancreatic duct cells *in vivo*, but conflicting results have left this question unanswered. Solar et al. demonstrated that adult Hnf1*β*+ cells do not differentiate into acinar or endocrine cells^12^. In addition, a cell-tracking experiment of adult Hes1+ cells found no evidence of acinar or endocrine differentiation^13^. However, these reports do not completely refute the existence of stem/progenitor cells in the adult ductal structure, because the expression of neither Hnf1*β* nor Hes1 represents the entire adult ductal epithelium. We have previously reported that Sox9 is expressed throughout the adult ductal tree and used in lineage-tracing experiments to demonstrate the continuous supply of new acinar cells from the adult Sox9-expressing ductal component in *Sox9-IRES-CreER* knock-in (*Sox9^CreERT2^*) mice^14^. Considering the pancreatic exocrine structure, centroacinar cells that localize at the tip of the Sox9-expressing ductal structure are thought to be the best candidate for acinar progenitors that function in keeping physiological homeostasis in *Sox9^CreERT2^* mice. However, another lineage-tracing experiment using BAC *Sox9-CreER* transgenic mice provided no evidence of acinar cell differentiation from adult Sox9+ cells^15^. Therefore, exploration of the mechanism by which new acinar cells are supplied from the Sox9-expressing cells in *Sox9^CreERT2^* mice should provide insights into the plasticity of adult pancreatic duct/centroacinar cells.

During embryonic stages, several transcription factors and signals control cell differentiation machineries in pancreas organogenesis^16^. For example, the amounts of expressed Sox9 and Ptf1a have been shown to influence the differentiation of endocrine and exocrine lineages, respectively^17^,^18^. In addition, many reports have revealed the pivotal role of Notch signaling in pancreas formation: overexpression of the Notch intracellular domain (NICD) suppresses endocrine and exocrine differentiation^19^,^20^,^21^, while inactivation of Hes1, the main effector of Notch signaling, causes inadequate expansion of pancreatic progenitors and early premature differentiation resulting in hypoplastic pancreas formation^22^,^23^,^24^. While the effect of the dosage of transcription factors such as Sox9 and Ptf1a has not been fully investigated in the adult organ, that pancreatic regeneration after cerulein-induced pancreatitis requires the reactivation of Notch signaling in mice supports the notion that Notch signaling is involved in controlling adult pancreatic cell plasticity^25^. In addition, Kopinke et al. reported that Hes1+ duct cells do not normally differentiate into acinar cells, but do exhibit rapid differentiation into the acinar cell type after inactivation of Rbpj in *Hes1-CreER* knock-in mice^13^,^26^.

In the present study, we aimed to analyze how the differentiation ability of Sox9+ cells into acinar cells is controlled in *Sox9^CreERT2^* mice. We revealed that Sox9 expression is decreased but that Hes1-mediated Notch signaling is normally conserved in the pancreas of adult *Sox9^CreERT2^* mice. Hes1-depletion accelerates acinar cell differentiation from Sox9-expressing duct cells in *Sox9^CreERT2^* mice, whereas NICD induction suppresses it. In addition, we show that Notch signaling positively regulates Sox9 and Hes1 in parallel. Based on these findings, we propose that the strength of Hes1-mediated Notch signaling and the dosage of Sox9 expression function cooperatively to control the plasticity of adult pancreatic duct cells.

## Results

### Pancreatic Sox9 expression is not altered in neonates but is reduced in adult Sox9-IRES-CreER knock-in mice

**Figure 1:**
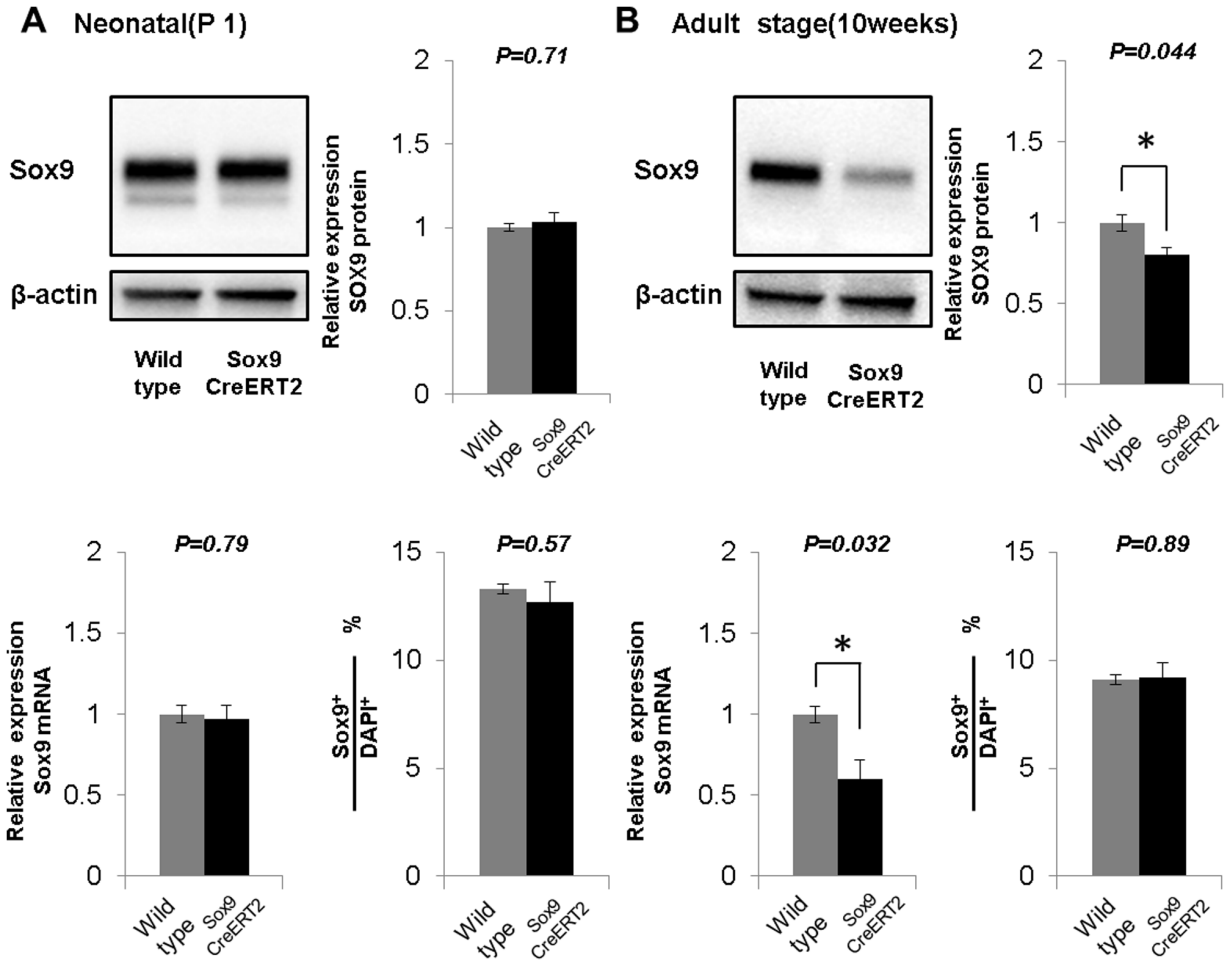
Reduced Sox9 expression in the adult pancreas of *Sox9^CreERT2^* mice. At the neonatal stage (A), the relative expression level of Sox9 in the pancreata of *Sox9^CreERT2^* mice is the same as that of wild-type mice as shown by Western blot (upper panels, n = 3 each) and qPCR (bottom left panel, n = 3 each). In the adult pancreas (B), Sox9 expression is less in *Sox9^CreERT2^* mice than in wild-type mice (Western blot shown in upper panels, n = 3 each and qPCR in bottom left panel, n = 3, 4). The ratio of Sox9-expressing cells among total DAPI+ epithelial cells is not significantly different between wild-type and *Sox9^CreERT2^* mice at P1 or the adult stage (bottom right panels in A and B, n = 3 each, refer to Supplementary Table S1). *P < 0.05. Data are expressed as means ± s.e.m.

In *Sox9^CreERT2^* mice, the *IRES-CreER* cassette is inserted in the 3′UTR of the Sox9 locus, thus the altered structure of the Sox9 locus potentially disrupts the control machinery of Sox9 expression in *Sox9^CreERT2^* mice^27^. At postnatal day 1 (P1), Western blotting and quantitative PCR analyses showed no difference in the expression of Sox9 between wild-type and *Sox9^CreERT2^* heterozygous mice (Fig. 1A). However, at the adult stage, Sox9 expression in *Sox9^CreERT2^* heterozygous mice was reduced to 80 ± 4.3% and 59 ± 12% of that in age-matched wild-type mice at the protein and mRNA level, respectively (Fig. 1B). The expression of Sox9 was restricted to CK19-expressing duct/centroacinar cells and the ratio of Sox9-expressing cells among total pancreatic epithelial cells was the same in wild-type and *Sox9^CreERT2^* mice both at the neonatal stage and adulthood (Fig. 1A, B bottom right panels and Supplementary Table S1), indicating that pancreatic duct/centroacinar cells in *Sox9^CreERT2^* mice express an equal amount of Sox9 in the newborn stage, but less in adulthood than do wild-type mice.

### Expression of Hes1 is the same in adult wild-type and Sox9-IRES-CreER knock-in mice

**Figure 2:**
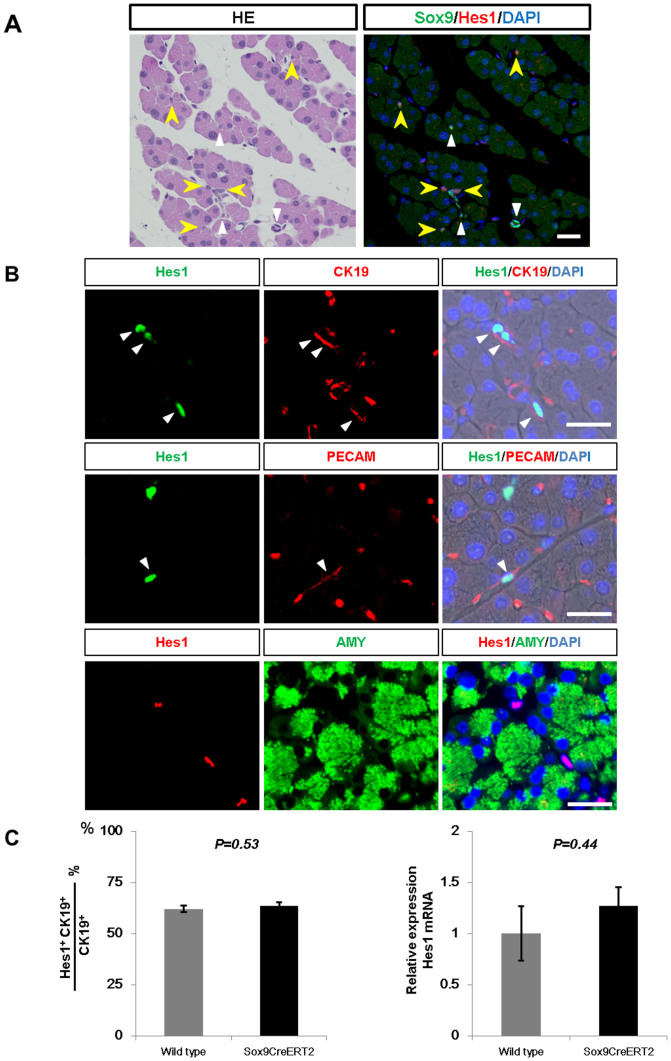
Unaltered Notch signal in adult *Sox9^CreERT2^* mice at steady state. (A) In the adult pancreas of *Sox9^CreERT2^* mice, Hes1 is expressed in a subpopulation of Sox9+ duct/centroacinar cells. White and yellow arrowheads indicate Sox9 single positive and Sox9/Hes1 double positive duct/centroacinar cells, respectively. The same section was used for both images. Scale bars = 25 μm. (B) Periodical expression of Hes1 in the ductal tree is confirmed by co-immunostaining of Hes1 and CK19 (upper panels). Note that Hes1 is expressed in a subset of Pecam-expressing endothelial cells (white arrowheads in the middle panels) but not in amylase+ acinar cells (bottom panels) in the adult pancreas of *Sox9^CreERT2^* mice. Scale bars = 25 μm. (C) In adult *Sox9^CreERT2^* mice, the ratio of Hes1+ cells to total CK19+ duct/centroacinar cells is unaltered compared with that of wild-type mice (left, n = 3 each, refer to Supplementary Table S2). qRT-PCR revealed that the Hes1 mRNA amount is the same in adult *Sox9^CreERT2^* and wild-type mice (right, n = 3 each). Data are expressed as means ± s.e.m.

Considering a previous report that showed the inactivation of Rbpj, a main effector of Notch signaling, causes rapid differentiation of adult Hes1-expressing duct/centroacinar cells into the acinar cell type^26^, we questioned if Notch activity is reduced in the adult pancreas of *Sox9^CreERT2^* mice and evaluated the activity by Hes1 expression, another main effector of Notch signaling. Consistent with previous findings on wild-type mice^13^,^15^,^28^, our immunohistochemical analyses revealed that Hes1 is periodically expressed in the Sox9+ ductal compartment, including the centroacinar cells in the adult pancreas of *Sox9^CreERT2^* mice (Fig. 2A). Quantitatively, up to 63.6 ± 1.57% of CK19+ duct/centroacinar cells were positive for Hes1 in *Sox9^CreERT2^* mice, which was not significantly different from wild-type mice (Fig. 2B, C and Supplementary Table S2). Hes1 expression was also observed in a subset of Pecam+ endothelial cells (Fig. 2B) and islets (data not shown), but we detected no Hes1 expression in amylase+ acinar cells (Fig. 2B). Quantitative PCR analyses showed no difference in Hes1 mRNA amounts between adult wild-type and adult *Sox9^CreERT2^* mice (Fig. 2C). Taken together, we concluded that Hes1 expression is reduced no more in the pancreas of adult *Sox9^CreERT2^* mice than it is in the pancreas of wild-type mice.

### Notch signaling controls the differentiation ability of Sox9+ duct/centroacinar cells into the acinar cell type in adult Sox9-IRES-CreER knock-in mice

**Figure 3:**
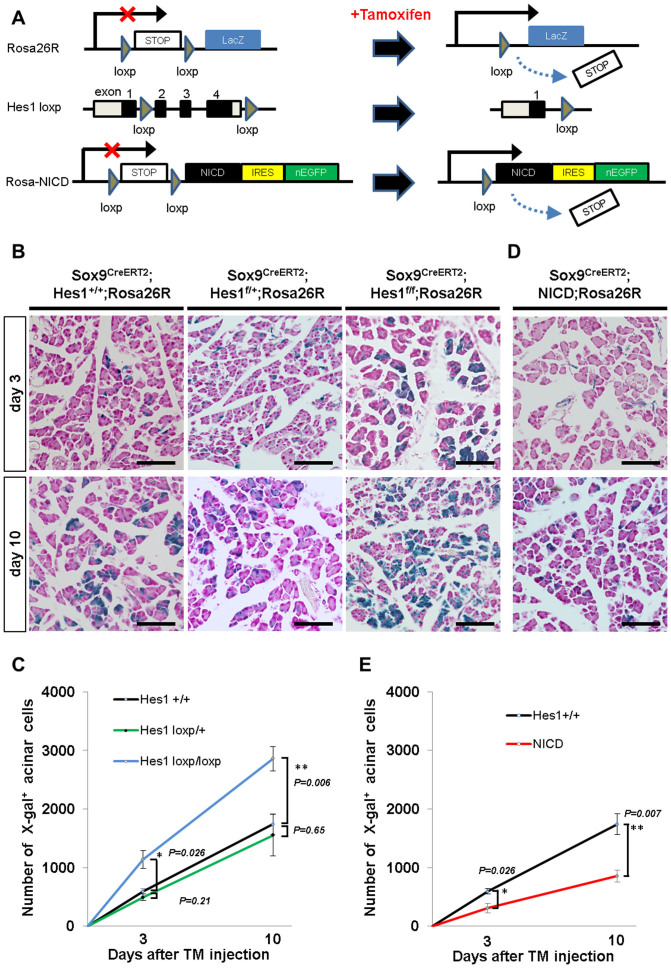
Notch signaling regulates the ability of duct-to-acinar differentiation in adult *Sox9^CreERT2^* mice. (A) Experimental strategy of tamoxifen-inducible Cre-mediated lineage tracing (upper panel) and Notch modulation in Sox9+ cells (middle panel, Hes1 depletion; lower panel, NICD induction). (B and D) Representative sections with X-gal staining 3 days and 10 days after single tamoxifen injection. X-gal-positive acinar cells, progenies of Sox9-expressing duct/centroacinar cells, increased from day 3 to day 10 in all the transgenic mice tested. (C and E) Quantitative analyses of the numbers of lineage-labeled acinar cells (refer to Supplementary Table S3). Note that conditional Hes1 depletion significantly accelerates acinar cell labeling in *Sox9^CreERT2^; Hes1^loxp/loxp^; Rosa26R* mice ((C), blue, P = 0.026 at day 3, P = 0.006 at day 10), but heterozygous inactivation (*Sox9^CreERT2^; Hes1^loxp/+^; Rosa26R* mice, shown in green) does not ((C), P = 0.21 at day 3, P = 0.65 at day 10). Activation of Notch signaling by NICD induction results in reduced acinar cell labeling in *Sox9^CreERT2^; Rosa^NICD^; Rosa26R* mice compared with *Sox9^CreERT2^; Hes1^+/+^; Rosa26R* control mice ((E), red, P = 0.026 at day 3, P = 0.007 at day 10). Scale bars = 100 μm. *P < 0.05, **P < 0.01. Data are expressed as means ± s.e.m.

We next questioned whether the modulation of Notch activity influences the differentiation ability of adult Sox9+ duct/centroacinar cells in *Sox9^CreERT2^* mice. For this purpose, we designed two sets of tamoxifen-inducible Cre-mediated experiments and evaluated the number of lineage-labeled acinar cells (Fig. 3A). First, we bred *Sox9^CreERT2^; Hes1^loxp/loxp^; Rosa26R* mice, in which Sox9+ cells have Hes1 null status due to tamoxifen-induced recombination. The second experiment inducibly forced the expression of NICD, which constitutively activates Notch signaling in Sox9+ cells from *Sox9^CreERT2^; Rosa^NICD^; Rosa26R* mice. In both experiments, tamoxifen treatment caused nuclear localization of the CreERT2 protein, and CreERT2-mediated recombination excised out the STOP cassette of the *Rosa26R* allele and activated lacZ expression to enable the detection of the recombined cells and their progeny as X-gal-positive cells (Fig. 3A). Without tamoxifen administration, no X-gal positive cells were detected in all experimental settings (Supplementary Fig. S1), thus the difference in the number of X-gal positive acinar cells is expected to represent the difference in the differentiation ability of lineage-labeled duct/centroacinar cells provided that both the *Rosa26R* allele and Hes1^loxP^ or NICD allele are recombined (see below).

As we have previously demonstrated^14^, the number of X-gal-positive acinar cells gradually increases after a single injection of tamoxifen (0.2 mg/g body weight) in *Sox9^CreERT2^; Hes1^+/+^; Rosa26R* mice, suggesting a continuous acinar cell supply from the Sox9-expressing duct/centroacinar cell compartment (Fig. 3B, C, E). While a 50% reduction of Hes1 dosage caused no apparent change in the number of labeled acinar cells in *Sox9^CreERT2^; Hes1^loxp/+^; Rosa26R* mice, conditional Hes1 depletion in *Sox9^CreERT2^; Hes1^loxp/loxp^*; *Rosa26R* mice resulted in accelerated acinar cell labeling (Fig. 3B, C). Quantitatively, the average number of X-gal-positive acinar cells was 1140 and 2859 at days 3 and 10 after tamoxifen injection, respectively, in *Sox9^CreERT2^; Hes1^loxp/loxp^; Rosa26R* mice, which was larger than the 594 cells and 1741 cells in control *Sox9^CreERT2^; Hes1^+/+^; Rosa26R* mice (Fig. 3C and Supplementary Table S3). On the contrary, forced expression of NICD caused a decrease in the number of lineage-labeled acinar cells (Fig. 3D, E), as the average number of X-gal-positive acinar cells in *Sox9^CreERT2^; Rosa^NICD^; Rosa26R* mice was 308 and 857 at days 3 and 10 (Fig. 3E and Supplementary Table S3).

Results of the lineage tracing experiments above indicate that Notch signaling negatively regulates the differentiation ability of Sox9-expressing progenitors into the acinar cell type in adult *Sox9^CreERT2^* mice. However, this conclusion requires further examination of whether lineage-labeled cells eventually depleted Hes1 and induced NICD expression in *Sox9^CreERT2^; Hes1^loxp/loxp^*; *Rosa26R* mice and *Sox9^CreERT2^; Rosa^NICD^; Rosa26R* mice, respectively. Since Hes1 is not expressed in acinar cells at steady state (Fig. 2B), it is impossible to dismiss the existence of lineage-labeled acinar cells that escaped the recombination of the Hes1^loxP^ allele in *Sox9^CreERT2^; Hes1^loxp/loxp^*; *Rosa26R* mice by immunostaining of Hes1. However, even if the existence of these escaper cells is taken into consideration, activated differentiation into the acinar cell type is supported by the following observation: Hes1/X-gal double positive cells were not detected in the duct/centroacinar compartment, whereas Hes1 expression was preserved in X-gal negative ducts at the same frequency as that in wild type or *Sox9^CreERT2^* mice without tamoxifen treatment (Fig. 2 and Supplementary Fig. S2A). In addition, the ratio of cell proliferation to cell death remained the same in *Sox9^CreERT2^; Hes1^loxp/loxp^*; *Rosa26R* mice (data not shown). In *Sox9^CreERT2^; Rosa^NICD^; Rosa26R* mice, we tested EGFP expression, which was co-induced with NICD by Cre-mediated activation of the *NICD-IRES-EGFP* cassette (Fig. 3A), and found that lineage-labeled acinar cells contained EGFP-negative cells (Supplementary Fig. S2B). Therefore, the results in Fig. 3E underestimate the impact of suppressed acinar differentiation by NICD induction. On the other hand, the existence of EGFP/X-gal double positive acinar cells indicates that NICD induction does not completely block the differentiation of duct/centroacinar cells into the acinar cell type in *Sox9^CreERT2^; Rosa^NICD^; Rosa26R* mice (Supplementary Fig. S2B). This notion was confirmed by the existence of Hes1/X-gal double positive acinar cells (Supplementary Fig. S2B). These findings indicate that activated Notch activity suppresses but does not completely block the acinar cell supply from the duct/centroacinar compartment in adult *Sox9^CreERT2^* mice, a property that contrasts the complete acinar cell agenesis by NICD induction during embryonic stages^19^,^20^.

It was reported that rapid differentiation into acinar cell types caused by Rbpj depletion in Hes1-expressing cells is accompanied by the loss of a centroacinar cell population^26^. However, Hes1 seems to be dispensable for the long-term maintenance of organ homeostasis in *Sox9^CreERT2^* mice, as centroacinar cells were not lost and the normal acinar structure was maintained in *Sox9^CreERT2^; Hes1^loxp/loxp^; Rosa26R* mice, suggesting the existence of a compensatory machinery. In fact, our lineage-tracing experiments using *Sox9^CreERT2^; Hes1^loxp/loxp^; Rosa26R* mice showed that both the duct and acinar cells retained their labeling status up to one year after tamoxifen injection (Supplementary Fig. S3A), indicating that Sox9-expressing duct/centroacinar cells could self-duplicate and continuously supply new acinar cells after Hes1 depletion. In addition, X-gal-labeled endocrine cells were not observed, indicating that Hes1 inactivation did not induce transdifferentiation of duct cells into endocrine cells in the adult pancreas (Supplementary Fig. S3B).

### Ptf1a expression is not influenced by the modulation of notch signaling in the adult pancreas

**Figure 4:**
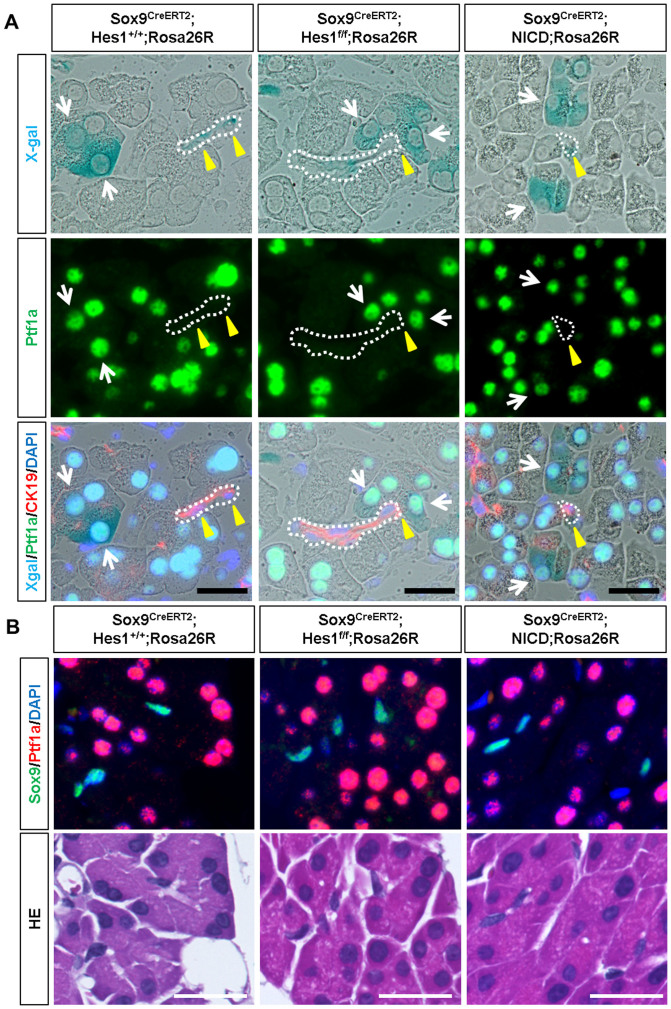
Ptf1a expression is not altered by Notch modulation in adult *Sox9^CreERT2^* mice. Expression of Ptf1a is confined to acinar cells and not affected by the modulation of Notch activity. (A) Ptf1a is expressed in lineage-labeled acinar cells (white arrows) and surrounding non-labeled acinar cells but not in CK19-expressing duct/centroacinar cells (dotted line and yellow arrowheads) in *Sox9^CreERT2^; Hes1^+/+^; Rosa26R* mice (left panels), *Sox9^CreERT2^; Hes1^loxp/loxp^; Rosa26R* mice (middle panels) and *Sox9^CreERT2^; Rosa^NICD^; Rosa26R* mice (right panels). The same section was used for X-gal staining and immunostaining in each column. Scale bars = 25 μm. (B) Ptf1a expression is confirmed by the co-staining of Ptf1a and Sox9 using sections obtained after high-dose tamoxifen treatment (refer to Supplementary Fig. S4). Scale bars = 25 μm.

While its role in the adult pancreas is still unknown, Ptf1a is indispensable for acinar cell differentiation^29^,^30^,^31^ and Notch signaling negatively regulates Ptf1a expression^22^,^32^,^33^ during pancreatogenesis. Considering the continuous acinar cell supply from the Sox9+ component in adult *Sox9^CreERT2^* mice, we questioned whether Ptf1a is ectopically expressed in Sox9+ cells, especially in centroacinar cells in adult *Sox9^CreERT2^* mice and whether modulation of Notch activity affects Ptf1a expression. However, as shown in Fig. 4A, Ptf1a expression is confined to acinar cells and not detected in the CK19-positive ductal structure, which includes centroacinar cells at steady state (*Sox9^CreERT2^; Hes1^+/+^; Rosa26R* mice). Additionally, it was affected by neither Hes1 depletion nor NICD induction, indicating that Notch-mediated Ptf1a suppression is less robust at adult stages than at embryonic stages. Indeed, Ptf1a expression was not detected in X-gal-positive duct cells of *Sox9^CreERT2^; Hes1^loxp/loxp^; Rosa26R* mice, confirming Hes1 depletion (Supplementary Fig. S2A). Furthermore, we treated mice with a higher dosage of tamoxifen (four injections of 0.2 mg/g body weight), which achieved more efficient labeling (approximately 60%, data not shown) of Sox9+ duct cells in control mice (Supplementary Fig. S4A). Efficient recombination was confirmed by observing a decrease and increase in the number of Hes1-expressing cells in *Sox9^CreERT2^; Hes1^loxp/loxp^; Rosa26R* mice and *Sox9^CreERT2^; Rosa^NICD^; Rosa26R* mice, respectively (Supplementary Fig. S4B). Again, we detected no ectopic Ptf1a expression, at least by immunostaining, in the Sox9+ ductal component of *Sox9^CreERT2^; Hes1^loxp/loxp^; Rosa26R* mice (Fig. 4B). In *Sox9^CreERT2^; Rosa^NICD^; Rosa26R* mice, Ptf1a expression was confined to the Sox9-negative acinar cells (Fig. 4B), including the Hes1-expressing acinar cells that were formed by NICD induction (Supplementary Fig. S5). Thus, the expression pattern of Ptf1a was unperturbed by modulating Notch activity in *Sox9^CreERT2^* mice, as neither Sox9/Ptf1a double positive cells nor structural alterations were observed.

### Notch signaling regulates Sox9 expression in adult pancreatic duct cells

**Figure 5:**
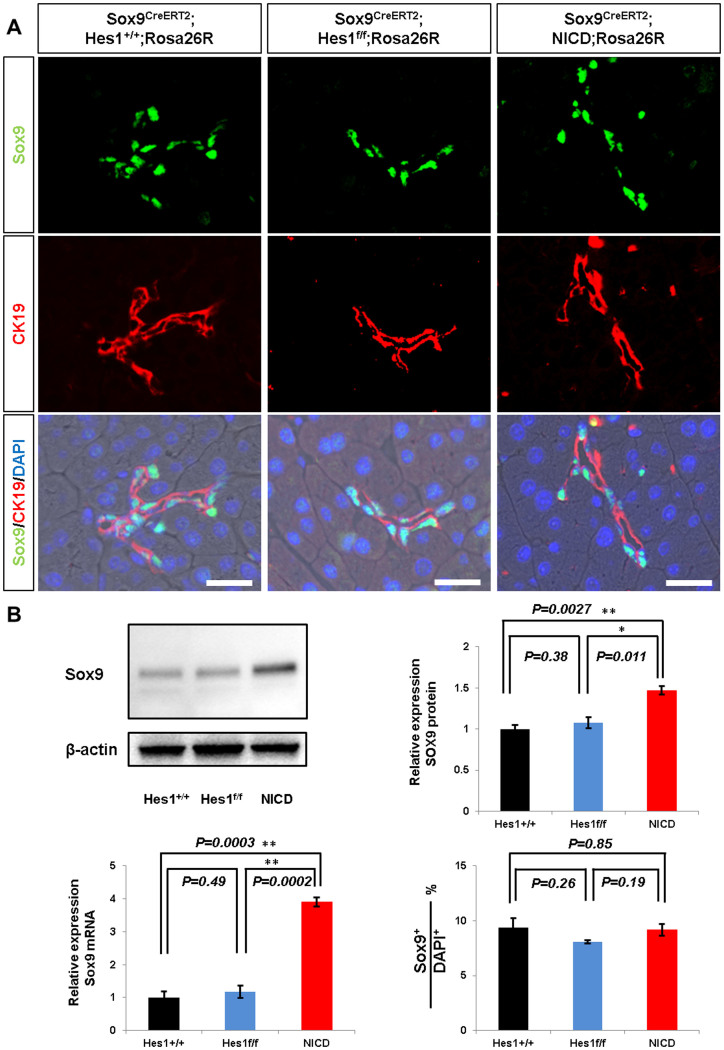
Notch signaling regulates Sox9 expression in adult *Sox9^CreERT2^* mice. Sox9 immunostaining, Western blotting, qPCR and cell counting were carried out after high-dose tamoxifen treatment (see Supplementary Fig. S4). (A) Neither Hes1 inactivation nor NICD induction causes ectopic Sox9 expression. Sox9 expression is confined to CK19+ duct/centroacinar cells. Scale bars = 25 μm. (B) Western blotting (upper panels) and the real-time qRT-PCR analyses (bottom, left panel) show that the expression level of Sox9 is increased by NICD induction (n = 3, P = 0.0027 and 0.0003, respectively) but not influenced by Hes1 inactivation (n = 3, P = 0.38 and 0.49, respectively). The ratio of Sox9+ cells to total DAPI+ epithelial cells is not altered by the modulation of Notch activity (bottom right panel, refer to Supplementary Table S4). *P < 0.05, **P < 0.01. Data are expressed as means ± s.e.m.

We found that the Sox9/CK19-expressing ductal component was structurally impaired by neither Hes1 depletion nor forced expression of NICD (Fig. 5A), even at the higher dosage of tamoxifen treatment (four injections of 0.2 mg/g body weight). Further, we did not detect any ectopic Sox9 expression in acinar cells, and the ratio of Sox9+ cells among total DAPI+ epithelial cells remained constant with modulating Notch activity (Fig. 5B and Supplementary Table S4). Notably, our Western blotting and quantitative PCR analyses revealed that the expression of Sox9 was elevated by the forced expression of NICD (Fig. 5B). On the other hand, Hes1 depletion did not influence the amount of Sox9 or the number of Sox9-expressing cells, reflecting the asymmetric division of Sox9+ cells into acinar and Sox9+ cells in *Sox9^CreERT2^* mice^14^. Thus, considering the increased number of Hes1-expressing cells by NICD induction (Supplementary Fig. S4 and S5), our findings show that Notch positively regulates Hes1 and Sox9 in parallel in adult pancreatic duct cells of *Sox9^CreERT2^* mice and that the regulation of Sox9 expression by Notch is mediated in a Hes1-independent manner in the adult pancreas.

### Hes1-depleted duct cells do not show endocrine neogenesis after pancreatic duct ligation

**Figure 6:**
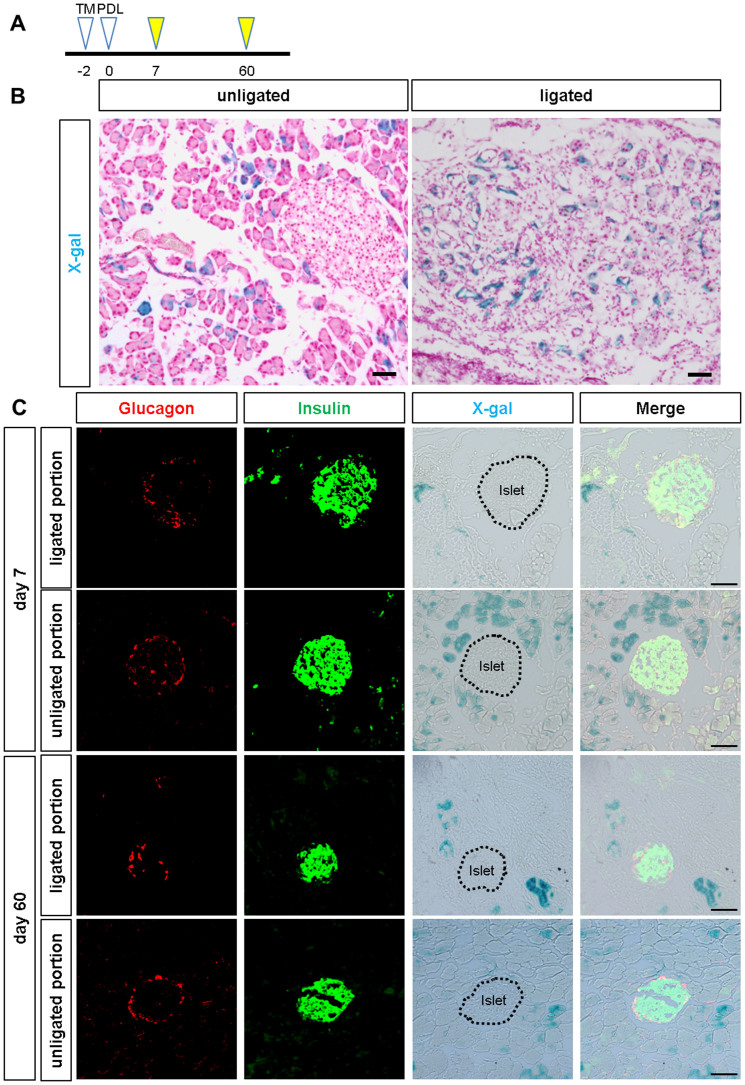
Hes1-deleted pancreatic duct cells do not contribute to islet neogenesis after PDL. (A) Schematic presentation of the experimental design. Two days before pancreatic duct ligation (PDL), a single injection of tamoxifen was given. Mice were analyzed 7 days and 60 days after PDL. (B) X-gal-stained pancreas section at 7 days after PDL. PDL induced acinar cell devastation and a duct-like structure in the ligated portion but not in the unligated portion. Scale bars = 50 μm. (C) Immunostaining revealed that neither insulin+ nor glucagon+ cells are labeled at 7 days or 60 days after PDL (n = 4 for day 7, n = 3 for day 60). Islets are encircled with dotted lines. Scale bars = 50 μm.

Another long-standing debate is whether adult pancreatic duct cells possess plasticity to differentiate into the endocrine lineage. In particular, possible islet neogenesis after pancreatic duct ligation (PDL) has attracted the interest of researchers^34^,^35^. We previously reported that beta cell neogenesis from Sox9-expressing duct cells was observed in neither the physiological condition nor the regeneration process after injury, including partial pancreatectomy, cerulein-induced acute pancreatitis, streptozotocin diabetes or PDL, in *Sox9^CreERT2^* mice^14^. Because Hes1 inactivation in the embryonic pancreas accelerates endocrine differentiation^23^, we tested whether Hes1 depletion combined with PDL prompts adult Sox9-expressing cells to differentiate into endocrine lineages in *Sox9^CreERT2^* mice. However, lineage tracing analyses detected no X-gal-positive cells in the islets at days 7 or 60 after PDL in *Sox9^CreERT2^; Hes1^loxp/loxp^; Rosa26R* mice (Fig. 6), indicating that adult Sox9-expressing duct/centroacinar cells are not capable of differentiating into endocrine lineages even by the combination of Hes1-inactivation and PDL.

## Discussion

In this study, we demonstrated that the plasticity of adult pancreatic duct cells is controlled by the strength of Notch signaling. The differentiation ability of Sox9-expressing duct/centroacinar cells into acinar cell types was accelerated by Hes1 inactivation and suppressed by NICD induction in *Sox9^CreERT2^* mice. This effect is consistent with two reports by Kopinke et al. that show Hes1-expressing centroacinar/terminal duct cells do not normally differentiate into acinar cells but will do so rapidly upon the inactivation of Rbpj in *Hes1-CreER* knock-in mice, which suggests that Notch signaling functions to maintain the duct cell identity in the adult pancreas^13^,^26^. Adding to that our previous report that showed Sox9-expressing duct/centroacinar cells continuously supply new acinar cells in adult *Sox9^CreERT2^* mice^14^, we hypothesized Hes1-negative/Sox9-positive centroacinar cells are best candidates for the origin of continuous acinar cell supply in *Sox9^CreERT2^* mice. However, Notch signaling alone cannot explain why Sox9-expressing cells were reported not to differentiate into acinar cells in adult BAC *Sox9-CreER* transgenic mice^15^. We therefore predicted the existence of one or more other mechanisms that control adult duct cell plasticity.

**Figure 7:**
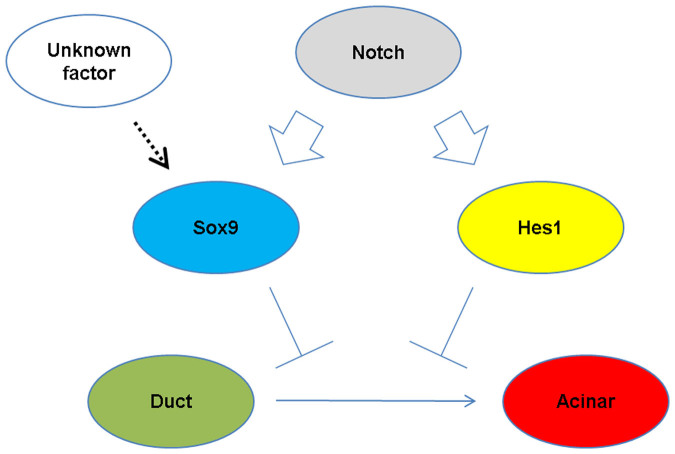
Proposed machinery for the regulation of duct-to-acinar differentiation in adult *Sox9^CreERT2^* mice. Notch upregulates Hes1 and Sox9 in parallel. Hes1 and Sox9 seem to function cooperatively to maintain the identity of the duct/centroacinar cell type. Continuous acinar differentiation from the duct/centroacinar cells in adult *Sox9^CreERT2^* mice correlates with reduced Sox9 expression. The existence of one or more unknown factors that regulate Sox9 expression in a Notch-independent manner is predicted.

We propose that the amount of Sox9 expression is pivotal in controlling adult duct cell plasticity (Fig. 7). The dosage requirement of Sox9 in the formation of the embryonic endocrine pancreas has been previously suggested by autopsy samples from three human cases of campomelic dysplasia, an autosomal dominant disorder caused by SOX9 haploinsufficiency that has been described as “less densely packed epithelial cells within the mesenchymal stroma and less clearly formed islets^36^.” The dosage requirement has also been experimentally supported previously; a Pdx1-Cre-mediated 50% reduction of Sox9 expression caused a decrease in the number of neurogenin 3 (Ngn3)-expressing endocrine progenitors and less endocrine formation in *Pdx1-Cre; Sox9^loxp/+^* mice^17^. However, in the human campomelic dysplasia cases, little attention was given to the exocrine tissue^36^, and exocrine pancreatic formation was not impaired in *Pdx1-Cre; Sox9^loxp/+^* mice^17^, suggesting that Sox9 dosage has less effect on exocrine pancreas formation during the embryonic stages. In contrast, our results suggest the involvement of Sox9 dosage in controlling the plasticity of adult pancreatic duct cells. In the present study, we demonstrated that pancreatic Sox9 expression in neonates was the same in wild-type and *Sox9^CreERT2^* mice, whereas adult pancreatic duct cells express less Sox9 in *Sox9^CreERT2^* mice than in adult wild-type mice. It should be noted that two previously reported lineage-tracing experiments using *Sox9^CreERT2^* and BAC *Sox9-CreER* transgenic mice are consistent in that embryonic Sox9+ duct cells were sufficiently plastic to differentiate into acinar and endocrine lineages, an ability that persisted until the perinatal stages up to one week after birth^14^,^15^. Provided that the amount of Sox9 expression in BAC *Sox9-CreER* transgenic mice was kept at the same level as that of wild-type mice at the newborn stage and adulthood, reduced Sox9 expression may allow duct cells to differentiate into acinar cells in adult *Sox9^CreERT2^* mice, whereas higher Sox9 expression in BAC *Sox9-CreER* transgenic mice possibly functions to maintain the duct cell identity, thereby preventing continuous differentiation into acinar cell types.

We also show that the induction of NICD caused elevated Sox9 expression, indicating that Sox9 expression is regulated by Notch signaling in adult *Sox9^CreERT2^* pancreata. This result is consistent with Shih et al., who reported that Notch signaling regulates Sox9 expression in the embryonic pancreas^37^. An *in vitro* tissue culture experiment using wild-type pancreas at e12.5 further showed that Hes1 expression was reduced by the addition of a low-dose γ-secretase inhibitor, while Sox9 expression was decreased at a higher dosage, indicating that Notch regulates Hes1 and Sox9 in parallel in embryonic pancreata. Nevertheless, we hypothesized that the reduced Sox9 expression in the adult pancreas of *Sox9^CreERT2^* mice at steady state is unconnected with Notch-mediated regulation. Notch activity seemed normal based on the ratio of Hes1-expressing cell numbers to CK-positive ductal epithelia and the amount of Hes1 mRNA was the same between wild-type and *Sox9^CreERT2^* mice. In *Sox9^CreERT2^* mice, the *IRES-CreERT2* cassette is inserted in the 3′UTR of the Sox9 locus^27^. We therefore speculate that the altered structure of the Sox9 locus is related to the reduction of Sox9 expression^38^ in the adult pancreas, though the precise machinery has yet to be revealed. Recent reports showed a conserved sequence among species very near the site of the *IRES-CreERT2* cassette insertion, suggesting the existence of a regulatory element in this region^39^,^40^. Because the Sox9 expression was normal at the perinatal stage in *Sox9^CreERT2^* mice, Notch-independent Sox9 regulation using this element, if it exists, would be silent until adulthood, when it maintains the Sox9 expression and thereby maintains the identity of the duct cell type. Future studies to dissect the complete regulatory machinery of Sox9 are required.

Islet neogenesis has long been believed to occur in the ductal compartment during the tissue restoration process, but we did not observe evidence of islet regeneration from Sox9-expressing cells in a PDL model. Xu et al. showed that the number of Ngn3-positive endocrine precursor cells increases after PDL^34^. Furthermore, in BAC *Sox9-CreER* transgenic mice, it was reported that *de novo* Ngn3-expressing cells are of duct cell origin, but that they do not express other pivotal transcription factors, such as Nkx6.1 and Pax6, and thus are unable to drive the entire differentiation program into endocrine lineage^15^. Consistent with this observation, Xiao et al. showed that Ngn3-expressing cells originating from the ducts after PDL do not differentiate into mature endocrine cells^41^.

Recent reports have focused more on the plasticity of adult acinar cells. Inducible Cre-mediated cell tracking using *Ptf1a-CreERTM* knock-in mice showed that adult acinar cells differentiate into endocrine cells and migrated into the islet structure after PDL^35^. Moreover, the plasticity of adult acinar cells has been experimentally demonstrated in the process of malignant transformation. The induction of oncogenic Kras^G12D^ in the adult murine acinar cell type caused PanIN formation via acinar-to-ductal metaplasia (ADM) and developed into pancreatic ductal adenocarcinoma, whereas Kras^G12D^ activation in the duct cell type did not^42^. Interestingly, Sox9/CPA1 double positive cells emerge during the transdifferentiation process of ADM, and simultaneous Sox9 depletion and Kras^G12D^ activation does not cause ADM in *Ptf1a^CreERTM^; LSL-Kras^G12D^; Sox9^floxed/floxed^; Rosa26^YFP^* mice^42^. In addition, our preliminary experimental results showed the emergence of Sox9/Ptf1a double positive cells in the tissue restoration after cerulein-induced acute pancreatitis. These findings suggest that cell plasticity might be connected to the co-expression of transcription factors that normally defines specific cell types: Sox9 in duct cells and Ptf1a in adult acinar cells. In fact, Sox9/Ptf1a double positive cells exist in the early embryonic pancreatic epithelium from which all pancreatic cell types originate^15^,^35^,^43^. To note, the Sox9/CPA1 double positive cells and Sox9/Ptf1a double positive cells mentioned above accompany tissue structure alterations such as ADM, tissue restoration after injury or embryonic organogenesis, whereas we observed no structural change or Sox9/Ptf1a double positive cells in the present study. So far, a precise mechanism that explains the relationship between the degree of cell plasticity with the co-expression of cell type-specific transcription factors and tissue construction is lacking. Further examination of duct-originated diseases, such as intraductal neoplasms, that accompany with tissue destruction may help reveal such a mechanism.

## Methods

### Mice

To obtain Hes1-conditional knock-out mice, *Hes1^loxp/+^* mice^44^, *Sox9^IRES-CreERT2^* mice^27^ and *Rosa26^LacZ^ (Rosa26R)* mice^45^ were interbred. *Rosa26^NICD^* mice^19^ and *Sox9^IRES-CreERT2^; Rosa26R* mice were crossed to produce *Sox9^IRES-CreERT2^; Rosa26^NICD^; Rosa26R* mice. The pancreatic duct ligation (PDL) procedure was performed as previously described^34^. All animal experiments were performed in accordance with the Kyoto University guidelines for animal experiments and approved by the animal research committee of Kyoto University.

### Tamoxifen injection

For lineage tracing, adult mice older than 8 weeks of age (approximately 25–30 g in body weight) were injected intraperitoneally with tamoxifen (Sigma T-5648) at 0.2 mg/g body weight. To get higher recombination efficiency, we injected tamoxifen at 0.2 mg/g body weight 4 times every other day.

### Tissue preparation for X-gal staining and paraffin section

X-gal staining and paraffin section were performed as previously described^14^.

### Immunofluorescence

The antigen retrieval procedure was performed with Target Retrieval Solution pH 6.0 (Dako), Target Retrieval Solution High pH 9.0 (Dako) or Proteinase K (Dako) in accordance with the manufacturer's instructions, followed by overnight incubation at 4°C with primary antibodies (Supplementary Table S5). The sections were washed in PBS and incubated for 60 min with secondary antibodies (Supplementary Table S6), followed by counterstaining with 4, 6-diamidino-2-phenylindole (DAPI). Immunostaining for Hes1 and Ptf1a required the use of the TSA amplification Kit (Perkin Elmar). Images were visualized using HS All-in-one Fluorescence Microscopy BZ-9000E (Keyence).

### Cell counting

We chose 20 fields at random and counted X-gal-positive acinar cells in each mouse. For short-term chase in the adult pancreas, we examined 20 fields per mouse to count the X-gal-positive acinar cells and averaged them. Cell numbers were counted by ×200 magnification in 40 fields (at adult stage) and 6 fields (at P1) using three sections per mouse and the ratio of Sox9+ cells among all DAPI+ epithelial cells was calculated. To determine the ratio of Hes1+ cells among CK19+ duct cells, cell numbers were counted by ×400 magnification in 6 fields per mouse. All values are shown as mean ± s.e.m. All error bars represent s.e.m. *P* values were calculated using an unpaired Student's *t* test (two-tailed); *P* < 0.05 was considered significant.

### RNA isolation and RT-PCR

Total RNA was extracted using the RNeasy Mini Kit (Qiagen). First-strand cDNA synthesis was performed using Rever Tra Ace qPCR RT Master Mix (Toyobo). Quantitative RT-PCR for Hes1 and Sox9 was performed with Taq Man and SYBR Green. Independent experiments were performed three times and confirmed. Data were normalized in relation to the expression of GAPDH and HPRT1. The Sox9 and Hes1 probes used were TaqMan Gene Expression Assay nos. Sox9 = Mm00448840 − m1, Hes1 = Mm01342805 − m1, GAPDH = Mm99999915 − g1 and HPRT1 = Mm01545399 − m1 (Applied Biosystems). For the SYBR Green ΔCT method, the Sox9 primer sequences were forward, AGGAAGCTGGCAGACCAGTA and reverse, TCCACGAAGGGTCTCTTCTC; and the GAPDH primer sequences were forward, GTGTTCCTACCCCCAATGTGT and reverse, ATTGTCATACCAGGAAATGAGCTT. Analysis was performed using the standard curve and comparative CT methods. *P* values were calculated using an unpaired Student's *t* test (two-tailed); *P* < 0.05 was considered significant.

### Western blotting

Whole pancreatic tissues were lysed in RIPA buffer (Thermo Scientific Pierce) in the presence of protease inhibitor (Roche). Obtained samples were electrophoresed in 10% SDS-PAGE gels and transferred to PVDF membrane (Bio-Rad). Membranes were blocked with 5% skim milk in TBS-T and incubated with anti-Sox9 antibody (Millipore 1:500) or anti-β-actin antibody (MBL 1:1000) overnight at 4°C. After washing, membranes were reacted with HRP-conjugated secondary antibodies (DAKO) at 1:2000 dilution. Chemiluminescence was detected with Immobilon Western horseradish peroxidase substrate (Thermo Scientific Pierce) and visualized with a charge-coupled device camera (Ez-capture). The intensity of the bands was quantified with imaging analysis software (Image J) using β-actin as an internal control. *P* values were calculated using an unpaired Student's *t* test (two-tailed); *P* < 0.05 was considered significant.

## Author Contributions

Y.K. and S.H. designed the study, analyzed the data and prepared the manuscript. S.H. performed the experiments. H.A. and R.K. generated the mice. K.F., M.H., Y.A., K.T., M.S., T.G., K.H., Y.N. and W.T. provided technical support and discussion. S.U. supervised the project.

## Supplementary Material

Supplementary Information Supplementary Information
