# Supplementary material for: Impact of Sox9 Dosage and Hes1-mediated Notch Signaling in Controlling the Plasticity of Adult Pancreatic Duct Cells in Mice
Source: Sci Rep. 2015 Feb 17;5:8518. doi: 10.1038/srep08518 (PMC4330537; doi:10.1038/srep08518)
Supplement: Supplementary Information [file srep08518-s1.pdf]

Impact of Sox9 Dosage and Hes1-mediated Notch Signaling in Controlling the Plasticity of Adult Pancreatic Duct Cells in Mice

Shinichi Hosokawa[1,3],Kenichiro Furuyama[1,3],
Masashi Horiguchi[1,3],Yoshiki Aoyama[1,3],Kunihiko Tsuboi[1,3],Morito Sakikubo[1,3]
Toshihiko Goto[1,3],Koji Hirata[1,3],Wataru Tanabe[3,5] ,Yasuhiro Nakano[3], Haruhiko Akiyama[2], Ryoichiro Kageyama[4], Shinji Uemoto[1] and Yoshiya Kawaguchi[3,*]

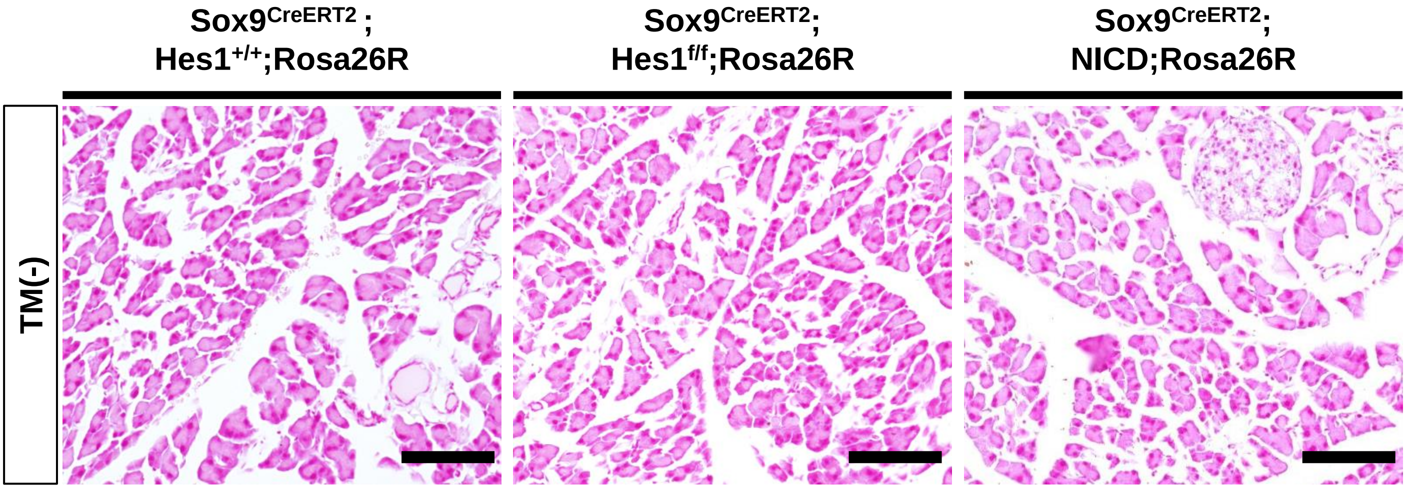

Supplementary Figure S1: Negative control experiment for Sox9 lineage-tracing study. Without tamoxifen treatment, X-gal positive cells are not detected in *$Sox9^{CreERT2}$; $Hes1^{+/+}$; Rosa26R*, *$Sox9^{CreERT2}$; $Hes1^{loxp/loxp}$; Rosa26R* and *$Sox9^{CreERT2}$; $Rosa^{NICD}$; Rosa26R*. Scale bars = 100 µm.

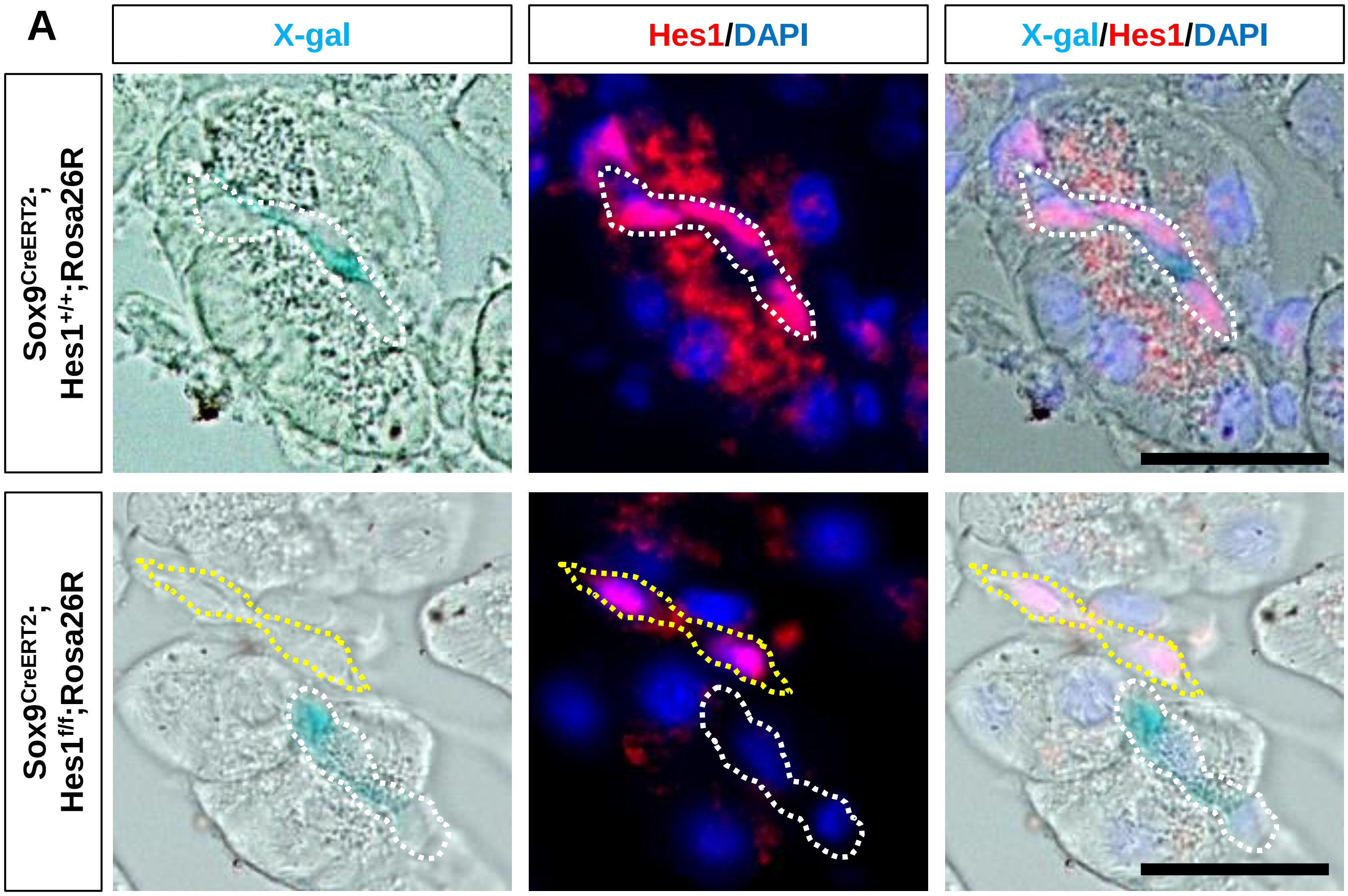

Supplementary Figure S2

A. Hes1 expression was completely deleted in lineage-labeled duct cells.

X-gal positive, lineage-labeled duct cells contain Hes1-expressing cells in *Sox9*^{CreERT2}*; Hes1*$^{+/+}$*; Rosa26R* mice (upper panels), whereas in *Sox9*^{CreERT2}*; Hes1*$^{loxp/loxp}$*; Rosa26R* mice, Hes1 expression was not detected in lineage-labeled duct cells (white dotted lines in bottom panels). Note that Hes1-expressing cells were conserved in X-gal^{-} ducts (bottom panels, yellow dotted lines) at the same frequency of that in wild type or *Sox9*^{CreERT2} mice without tamoxifen treatment . Scale bars = 25 μm.

B. Lineage-labeled acinar cells contain Notch-activated and non-activated cells in *Sox9*^{CreERT2}*; Rosa*^{NICD}*; Rosa26R* mice.

In *Sox9*^{CreERT2}*; Rosa*^{NICD}*; Rosa26R* mice, X-gal positive acinar cells are composed of EGFP-positive and negative populations, the former indicating successful recombination of both Rosa26R and Rosa NICD-IRES-EGFP alleles (compare the absence of X-gal and EGFP-double positive cells in the upper and middle panels). Activated Notch signaling by NICD induction was confirmed by X-gal, Hes1 double positive acinar cells (bottom panels). Yellow arrows indicate EGFP^{+}/X-gal^{+} or Hes1^{+}/X-gal^{+} acinar cells. White arrows indicate EGFP^{-}/X-gal^{+} or Hes1^{-}/X-gal^{+} acinar cells. Scale bars = 25 μm.

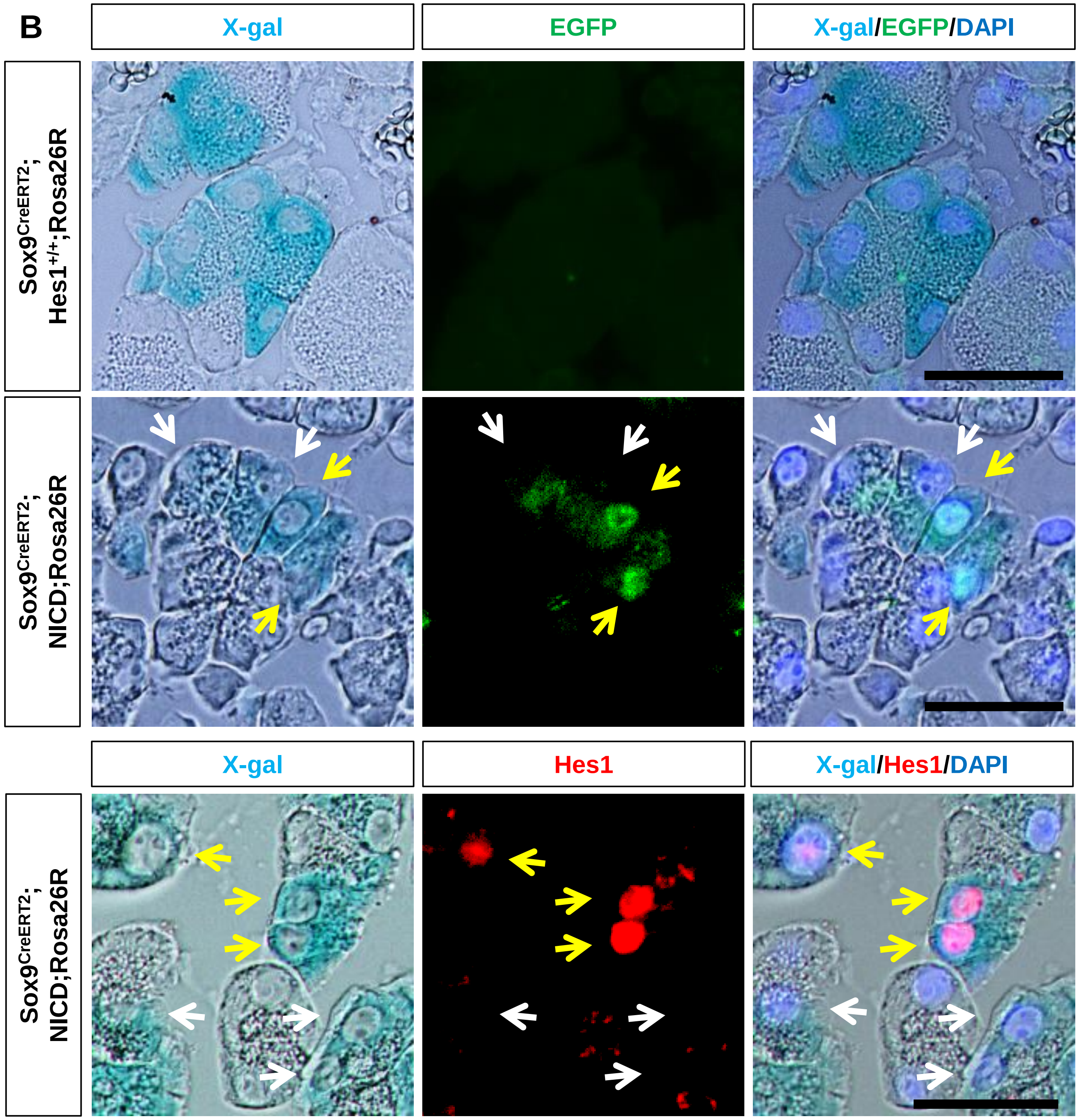
B
X-gal
EGFP
X-gal/EGFP/DAPI
Sox9CreERT2;
Hes1+/+;Rosa26R
Sox9CreERT2;
NICD;Rosa26R
X-gal
Hes1
X-gal/Hes1/DAPI
Sox9CreERT2;
NICD;Rosa26R

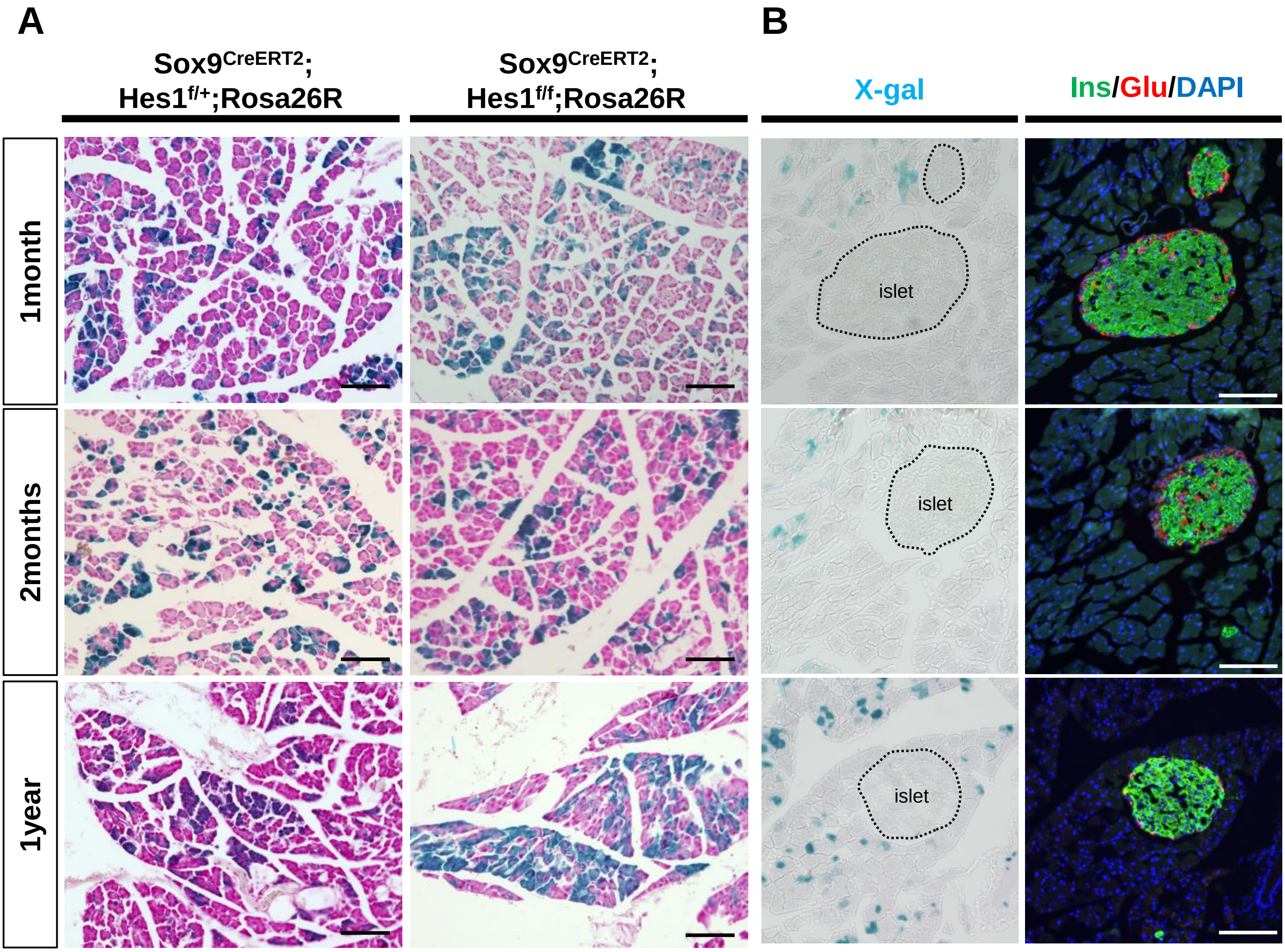

Supplementary Figure S3: Hes1 is dispensable for the maintenance of progenitor population.

(A) Long-term chase at 1 month, 2 months and one year after single tamoxifen injection. As time proceeds, lineage-labeled acinar cells gradually expanded in number in both *Sox9*^{CreERT2}*; Hes1*$^{loxp/+}$*; Rosa26R* and *Sox9*^{CreERT2}*; Hes1*$^{loxp/loxp}$*; Rosa26R* mice (representative pictures are shown). Notably, even one year after tamoxifen injection, pancreatic duct cells retain their X-gal labeled status and X-gal positive acinar cells are broadly spread, suggesting that Hes1 is dispensable for the self-duplication of Sox9-expressing progenitor cells and differentiation into the acinar cell type. (B) No X-gal-labeled cells are observed in insulin+ (green) or glucagon+ (red) endocrine cells even in the long-term chase periods. Scale bars = 100 μm.

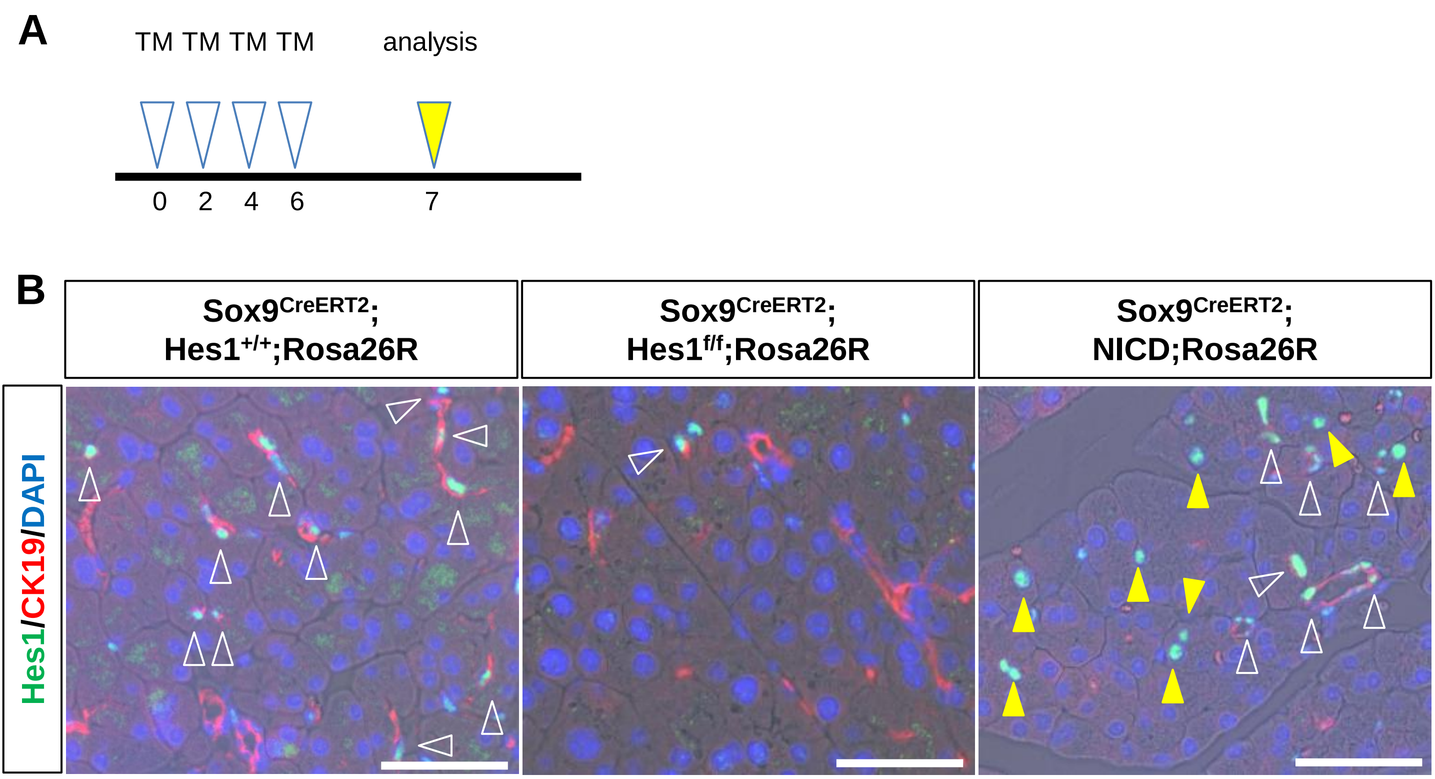

Supplementary Figure S4: **Modulation of Notch activity by high-dose tamoxifen treatment.** (A) To get more efficient recombination, tamoxifen was injected four times. (B) High dose tamoxifen treatment resulted in a decrease and increase in the number of Hes1-expressing cells in *Sox9^{CreERT2}; Hes1$^{loxp/loxp}$; Rosa26R* and *Sox9^{CreERT2}; Rosa^{NICD}; Rosa26R* mice, respectively. White and yellow arrowheads indicate Hes1^{+} duct/centroacinar and Hes1^{+} acinar cells, respectively. Scale bars = 50 μm.

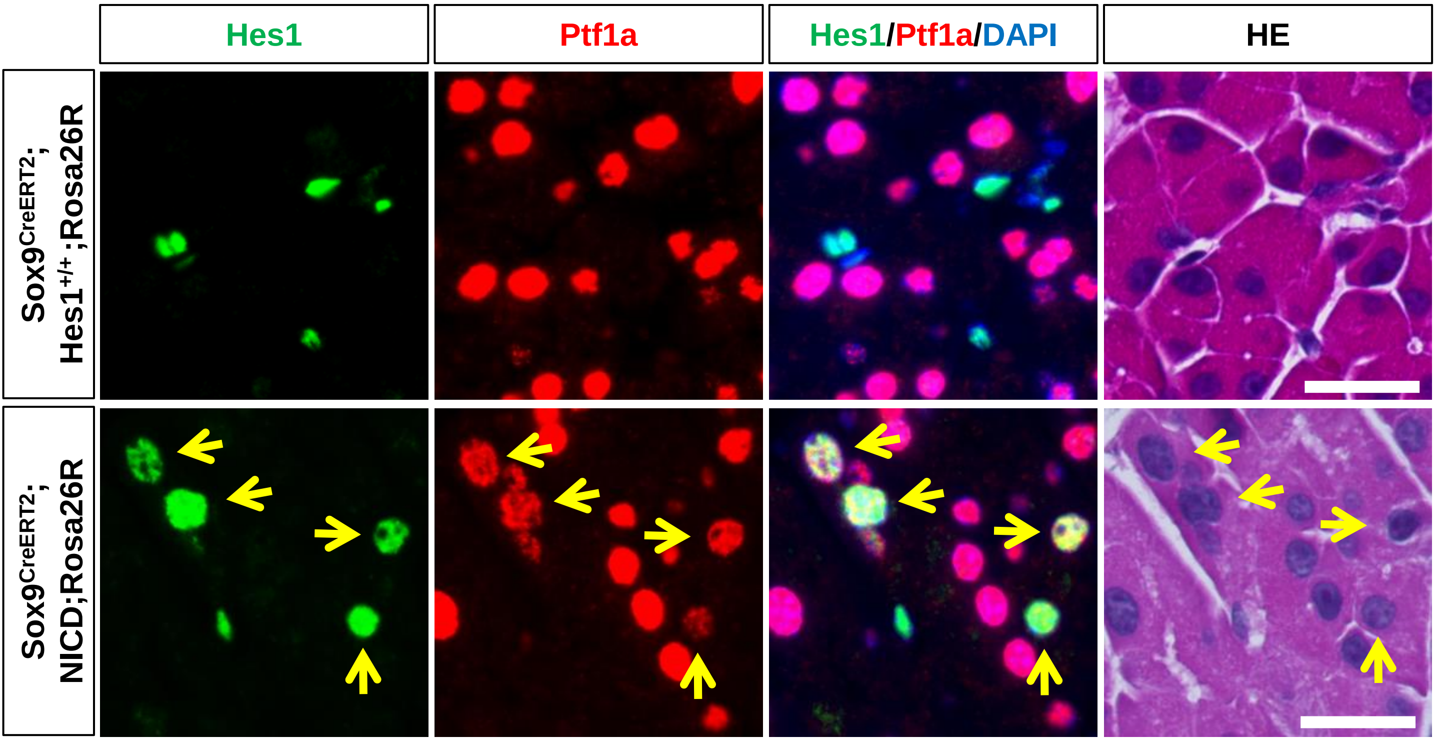

Supplementary Figure S5: **Emergence of Hes1/Ptf1a double positive acinar cells by NICD induction.**
In *Sox9*^{CreERT2}; *Rosa*^{NICD}; *Rosa26R* mice (lower panels), acinar cells contain Hes1/Ptf1a double positive cells (yellow arrows) which were not detected in the control mice (upper panels).
Scale bars = 25 μm.

Supplementary Table1

The ratio of Sox9 positive cells among all epithelial cells (% of total DAPI$^+$ cells)

Genotype		n	% of total epithelial cells	
Wild type	*P1*	*3*	*13.3±0.23*	(Sox9$^+$ / DAPI$^+$: 2634/19707)
Sox9^{CreERT2}	*P1*	*3*	*12.7±0.97*	(Sox9$^+$ / DAPI$^+$: 2690/21756)
Wild type	*adult*	*3*	*9.11±0.22*	(Sox9$^+$ / DAPI$^+$: 8390/92352)
Sox9^{CreERT2}	*adult*	*3*	*9.22±0.68*	(Sox9$^+$ / DAPI$^+$: 8138/88064)

The numbers shown represent the means number ± standard error of the means(s.e.m)

Supplementary Table2

Hes1 co-localization among CK19$^+$ ductal population (% of total CK19$^+$ cells counted)

Adult pancreas			
**Genotype**	**n**	**% of total duct cells**	
Wild type	***3***	***62.1±1.62***	**(Hes1$^+$CK19$^+$ / total CK19$^+$: 647 /1122)**
Sox9^{CreERT2}	***3***	***63.6±1.57***	**(Hes1$^+$CK19$^+$ / total CK19$^+$: 693 /1084)**

The numbers shown represent the means number ± standard error of the means(s.e.m)

Supplementary Table3

Number of lineage labeled acinar cells counted

Genotype	X-gal$^+$ acinar cells/20fields	average
**day 3**		
Sox9^{CreERT2};Hes1$^{+/+}$;Rosa26R	**664**	
Sox9^{CreERT2};Hes1$^{+/+}$;Rosa26R	**609**	**594**
Sox9^{CreERT2};Hes1$^{+/+}$;Rosa26R	**511**	
Sox9^{CreERT2};Hes1$^{loxp/loxp}$;Rosa26R	**709**	
Sox9^{CreERT2};Hes1$^{loxp/loxp}$;Rosa26R	**1188**	**1140**
Sox9^{CreERT2};Hes1$^{loxp/loxp}$;Rosa26R	**1250**	
Sox9^{CreERT2};Hes1$^{loxp/loxp}$;Rosa26R	**1413**	
Sox9^{CreERT2};Hes1$^{loxp/+}$;Rosa26R	**332**	
Sox9^{CreERT2};Hes1$^{loxp/+}$;Rosa26R	**520**	
Sox9^{CreERT2};Hes1$^{loxp/+}$;Rosa26R	**531**	**492**
Sox9^{CreERT2};Hes1$^{loxp/+}$;Rosa26R	**588**	
Sox9^{CreERT2};Rosa^{NICD};Rosa26R	**220**	
Sox9^{CreERT2};Rosa^{NICD};Rosa26R	**510**	**308**
Sox9^{CreERT2};Rosa^{NICD};Rosa26R	**355**	
Sox9^{CreERT2};Rosa^{NICD};Rosa26R	**146**	
**day 10**		
Sox9^{CreERT2};Hes1$^{+/+}$;Rosa26R	**1811**	
Sox9^{CreERT2};Hes1$^{+/+}$;Rosa26R	**1913**	**1741**
Sox9^{CreERT2};Hes1$^{+/+}$;Rosa26R	**1231**	
Sox9^{CreERT2};Hes1$^{+/+}$;Rosa26R	**2011**	
Sox9^{CreERT2};Hes1$^{loxp/loxp}$;Rosa26R	**2800**	
Sox9^{CreERT2};Hes1$^{loxp/loxp}$;Rosa26R	**3402**	**2859**
Sox9^{CreERT2};Hes1$^{loxp/loxp}$;Rosa26R	**2393**	
Sox9^{CreERT2};Hes1$^{loxp/loxp}$;Rosa26R	**2841**	
Sox9^{CreERT2};Hes1$^{loxp/+}$;Rosa26R	**779**	
Sox9^{CreERT2};Hes1$^{loxp/+}$;Rosa26R	**2262**	**1551**
Sox9^{CreERT2};Hes1$^{loxp/+}$;Rosa26R	**2035**	
Sox9^{CreERT2};Hes1$^{loxp/+}$;Rosa26R	**1131**	
Sox9^{CreERT2};Rosa^{NICD};Rosa26R	**1138**	
Sox9^{CreERT2};Rosa^{NICD};Rosa26R	**672**	**857**
Sox9^{CreERT2};Rosa^{NICD};Rosa26R	**774**	
Sox9^{CreERT2};Rosa^{NICD};Rosa26R	**846**	

Supplementary Table4

The ratio of Sox9 positive cells among all epithelial cells (% of total DAPI$^+$ cells)

**Adult pancreas**			
**Genotype**	**n**	**% of total epithelial cells**	
Sox9^{CreERT2}	***3***	***9.39±0.83***	**(Sox9$^+$ / DAPI$^+$: 7268/75825)**
Sox9^{CreERT2} ;Rosa^{NICD}	***3***	***9.18±0.54***	**(Sox9$^+$ / DAPI$^+$: 9118/99306)**
Sox9^{CreERT2} ;Hes1$^{loxp/loxp}$	***3***	***8.09±0.17***	**(Sox9$^+$ / DAPI$^+$: 7938/98487)**

The numbers shown represent the means number ± standard error of the means(s.e.m)

Supplementary Table 5 Primary Antibodies

Antigen	Species	Dilution	Supplier
Amylase	Rabbit	1:1000	Sigma
Amylase	Goat	1:100	Santa Cruz
CK19	Rat	1:500	DSHB
Glucagon	Rabbit	1:400	DAKO
GFP	Chicken	1:500	abcam
Hes1	Rabbit	1:5000	Dr.Sudo
Insulin	Guinea Pig	1:400	DAKO
PECAM-1	Rat	1:20	BD Biosciences
Ptf1a	Rabbit	1:10000	Dr.Hoshino
Sox9	Rabbit	1:1000	Millipore
Sox9	Goat	1:50	Santa Cruz

Supplementary Table 6 Secondary Antibodies

Conjugate	Antigen	Species	Dilution	Supplier
Alexa Fluor488	Mouse IgG	Goat	1:1000	Molecular Probes
Alexa Fluor488	Rabbit IgG	Donkey	1:1000	Molecular Probes
Alexa Fluor488	Guinea pig IgG	Goat	1:1000	Molecular Probes
Alexa Fluor488	Goat IgG	Donkey	1:1000	Molecular Probes
Cy3	Rat IgG	Donkey	1:500	Chemicon
Cy3	Rabbit IgG	Donkey	1:500	Chemicon
